# Preface

**DOI:** 10.1155/S0962935194000347

**Published:** 1994

**Authors:** Peter Vadas, Waldemar Pruzanski


					Preface
Mediators of Inflammation 3, 241 (1994)
Preface
A symposium entitled 'Novel Molecular Approaches
to Anti-inflammatory Therapy' will be held in To-
ronto, Canada on July 22-23, 1994. This symposium
organized under the auspices of the International
Association of Inflammation Societies (IAIS), will be
a satellite to the XlIth International Congress of
Pharmacology in Montreal, Canada.
The Inflammation Satellite will provide a 2 day
forum for presentation of 'cutting-edge' advances in
the field of anti-inflammatory therapy. These ad-
vances are now being used as the basis for a new
generation of therapeutics, and this satellite sympo-
sium will focus on recent advances in the field of
cytokine actions, inducible phospholipases A2 and
cyclooxygenase-2, cell adhesion events and novel
anti-inflammatory therapies. Five scientific sessions
will include:
I. Cytokines and Chemokines
II. Phospholipase A,. and cyclooxygenase-2
III.
IV.
V.
Cell adhesion molecules
Molecular approaches to new therapies
Poster session
Scientific presentations by 20 invited speakers will be
published as a monograph under separate cover. In
this issue of Mediators ofInflammation are included
the abstracts to be presented at the Poster session
Peter Vadas, MD, Phl)
Chairman,
Organizing Committee
IUPHAR Satellite on Inflammation
Waldemar Pruzanski MD
Chairman,
Publication Committee
IUPHAR Satellite on Inflammation
(C) 1994 Rapid Communications of Oxford Ltd Mediators of Inflammation Vol 3 1994 241

				

